# 

**Published:** 1901-09

**Authors:** 


### ASSOCIATION MEETINGS.

### SEPTEMBER.

### AMERICAN VETERINARY MEDICAL ASSOCIATION.

Atlantic City, September 3d, 4th, and 5th.

### INDIANA STATE VETERINARY ASSOCIATION.

In Indianapolis in September. DR. G. H. ROBERTS,
214 N. MERIDIAN ST., INDIANAPOLIS, IND. Secretary.

### SOCIETY OF VETERINARY GRADUATES OF WISCONSIN.

The semi-annual meeting of the Society of Veterinary Graduates of Wisconsin will be held in Milwaukee during the second week of September. DR. W. G. CLARK,
MARINETTE, WIS. Secretary.

### PENNSYLVANIA STATE VETERINARY MEDICAL ASSOCIATION.

The semi-annual meeting will be held in Pittsburg, September 17th.

### NEW YORK STATE VETERINARY MEDICAL SOCIETY.

The annual meeting will be held at Ithaca on September 10th and 11th. An elaborate and valuable programme is offered in original papers and clinical work. For details see August JOURNAL, page 535, or write to Dr. W. L. Williams, Ithaca, N. Y.

### WEST VIRGINIA VETERINARY MEDICAL ASSOCIATION.

The West Virginia Veterinary Medical Association will hold its meeting, September 2, 1901, at the Hotel Rudolf, Atlantic City, N. J.

### MASSACHUSETTS VETERINARY MEDICAL ASSOCIATION.

This Association will meet on September 25th, at 8 Fenway, Boston, Mass. Subsequent meetings on the fourth Wednesday of each month at the same place. DR. E. T. HARRINGTON,
SOUTH BOSTON, MASS. Secretary.

### NEBRASKA STATE VETERINARY ASSOCIATION.

Next regular meeting at Omaha, Neb., in the latter part of September. A. F. PETERS,
LINCOLN, NEB. Secretary.

OCTOBER.

TENNESSEE VETERINARY MEDICAL ASSOCIATION.

Next meeting at Nashville.

JOSEPH PLASKETT,
NASHVILLE, TENN. Secretary.

MAINE VETERINARY MEDICAL ASSOCIATION.

Next regular meeting at Lewiston, October 9th.

F. E. FREEMAN,
ROCKLAND, ME. Secretary.

MISSOURI VETERINARY MEDICAL ASSOCIATION.

The tenth annual meeting will be held at Kansas City, Missouri, early in October.

B. F. KAUPP,
3725 MICHIGAN AVE., KANSAS CITY, MO. Secretary.

---

PENNSYLVANIA STATE BOARD OF VETERINARY MEDICAL EXAMINERS,

### PERSONALS.

Dr. W. H. Yingst, of Harrisburg, Pa., is on a trip to South Africa with a shipload of mules for the English Government.

Dr. Alexander Glass, of Philadelphia, has resumed his daily trips along the Delaware River and Bay with his fast sailing yacht.

Dr. Oscar Seeley, of Philadelphia, was an exhibitor in the saddle classes at the Atlantic City Horseshow.

Dr. H. M. Hunter, of Visalia, Cal., is veterinarian for Tulare County, and gives special attention to veterinary sanitary police work.

Dr. Enoch H. Moore, formerly of Philadelphia, is now engaged in large commercial enterprises covering cities between St. Louis and Atlantic City.

The Department of Agriculture of New Hampshire has established at Durham a Department of Veterinary Medicine and Dairying. Marion Imes fills the rôle of assistant to this department.

Dr. Leonard Pearson, Dean of the Veterinary Department of the University of Pennsylvania, was in the State legislative halls at the final fall of the gavel.

Dr. J. Desmond, of Adelaide, South Australia, has just returned from Cape Town, South Africa, at which point he made an extended trip.

Dr. W. H. Knight, of Kennett Square, Pa., met with a serious loss by fire in the latter part of June.

Drs. A. D. Melvin, of Washington, D. C., and Charles Schaufler, of Philadelphia, representing the Bureau of Animal Industry, were Atlantic City visitors early in July.

Dr. Jesse Z. Hillegas, of Red Hill, Pa., has several fast pacemakers entered in the Grand Circuit.

Dr. C. K, Gary, of Hornellsville, N. Y., takes an active interest in trotting horses, his livery and boarding stables containing a number of good horses.

Dr. J. C. McNeil, city veterinarian of Pittsburg, Pa., was on the sick list for several days in June.

Dr. A. H. Fehr, of Canandaigua, N. Y., is the inventor and proprietor of the safety yoke for cattle.

Dr. Frank Standen, of Philadelphia, Pa., spent a month among the springs of Maine in July and August.

Dr. James T. Shannon, of Lexington, Ky., was a visitor to Philadelphia in June.

Dr. Leonard Pearson, State Veterinarian of Pennsylvania, left for Europe on August 1st to visit the leading veterinary colleges.

Dr. Oscar Seeley, of Philadelphia, was the winner of several ribbons at the Atlantic City Horseshow.

Dr. S. C. Rudd, of Woodstock, Ontario, a graduate of the Ontario Veterinary College, is one of the most enthusiastic horsemen in that part of Canada.

Dr. M. E. Conard, of West Grove, Pa., is chairman of the Institute Committee for Chester County in connection with the Department of Agriculture of Pennsylvania.

Dr. John Wende, of Buffalo, N. Y., is chief veterinarian of the Pan-American Exposition at Buffalo, N. Y.

Dr. T. A. Donaldson, of Manchester, Conn., after a long period of illness, is now very much improved.

Dr. R. L. Kann, of Mechanicsburg, Pa., who recently was licensed by the Pennsylvania State Board of Veterinary Medical Examiners, made a trip to South Africa on one of the English transports with a consignment of horses and mules.

Dr. G. E. Nesom, of the South Carolina Experiment Station, spends many hours among the Farmers' Institutes of the "Palmetto State."

Dr. E. M. Ranck, of the H. K. Mulford Company laboratories of Philadelphia, made a trip to the West and Southwest as far as New Orleans in August.

Dr. W. S. Corlis will succeed to the practice of Dr. John A. Bell, at Watertown, N. Y. Dr. Bell fills the rôle of United States Government Inspector at Watertown.

Dr. H. D. Martin, of Buffalo, N. Y., is assistant veterinarian to the Pan-American Exposition at Buffalo, N. Y.

Dr. Oscar Seeley's "Bon Ton" saddle horse is displayed on the pages of the *Breeders' Gazette*, August 7th issue.

Dr. James A. Waugh, of Pittsburg, will present a paper at the September meeting of the Pennsylvania State Veterinary Medical Association on "Alkalimetry in Veterinary Practice" and participate in the discussion of "Intestinal Obstructions in the Dog."

Dr. J. P. Foster, of Selby, South Dakota, takes a keen interest in matters Masonic, and holds a high place in the thirty-second degree Masonry.

Dr. T. B. Pote, formerly of Indiana, is now a resident of St. Louis, Mo.

Dr. W. P. Phipps, of Wilkesbarre, is the owner of "Lord Middleton," by "Star Wilkes," a fast trotting gelding.

